# Supplementary figures and images for: The carboxyl-terminal sequence of bim enables bax activation and killing of unprimed cells
Source: eLife. 2020 Jan 24;9:e44525. doi: 10.7554/eLife.44525 (PMC6980855; doi:10.7554/eLife.44525)

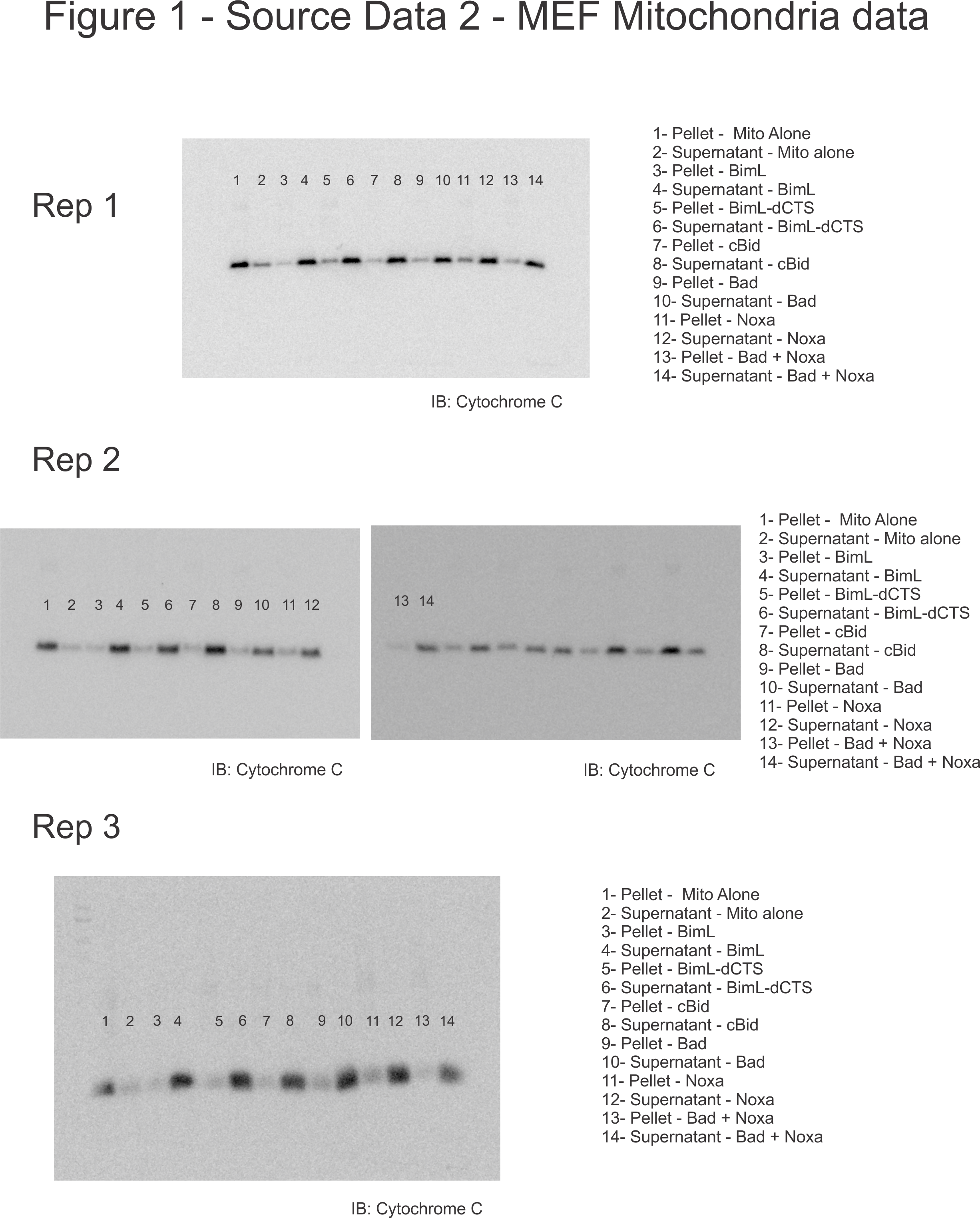

Supplement: Figure 1—source data 2. [file elife-44525-fig1-data2.zip › Figure 1 - Source Data 2.tif]

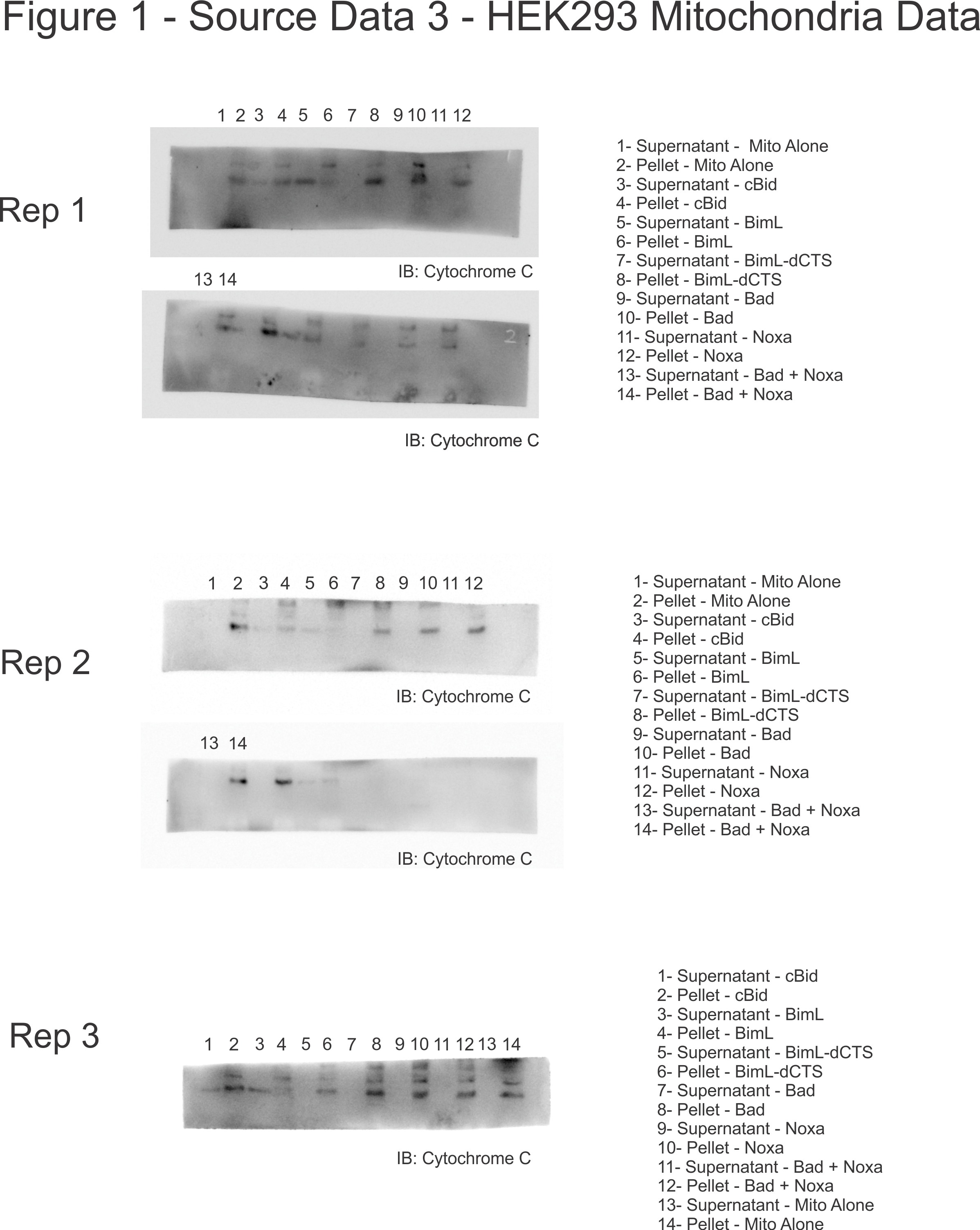

Supplement: Figure 1—source data 3. [file elife-44525-fig1-data3.zip › Figure 1 - Source Data 3.tif]

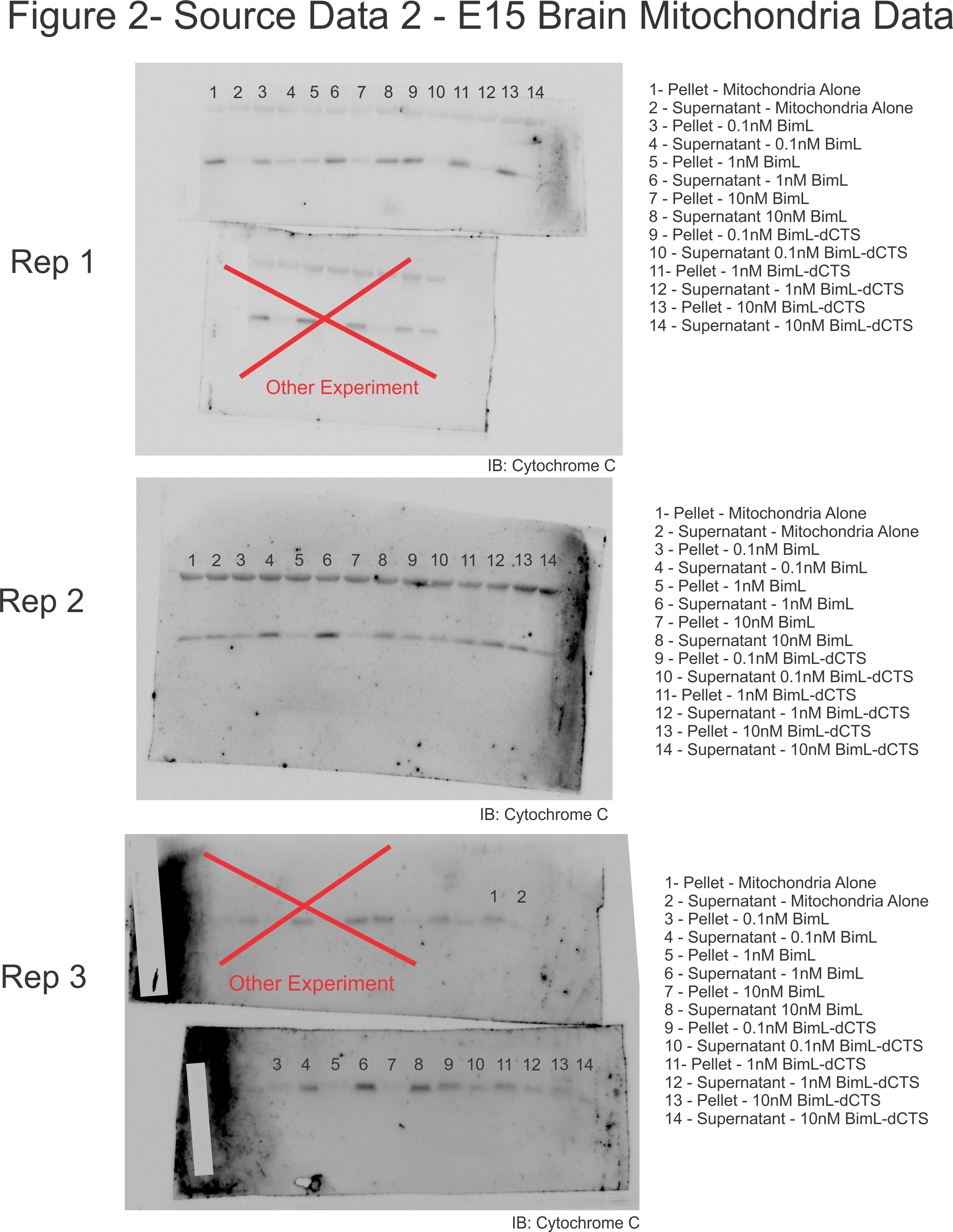

Supplement: Figure 2—source data 2. [file elife-44525-fig2-data2.zip › Figure 2 - Source Data 2.tif]
