# Supplementary material for: The carboxyl-terminal sequence of bim enables bax activation and killing of unprimed cells
Source: eLife. 2020 Jan 24;9:e44525. doi: 10.7554/eLife.44525 (PMC6980855; doi:10.7554/eLife.44525)
Supplement: Transparent reporting form [file elife-44525-transrepform.pdf]

eLIFE

1st Floor
24 Hills Road
Cambridge CB2 1JP, UK

P 01223 855340
W elifesciences.org
T @elife

eLife's transparent reporting form

We encourage authors to provide detailed information *within their submission* to facilitate the interpretation and replication of experiments. Authors can upload supporting documentation to indicate the use of appropriate reporting guidelines for health-related research (see EQUATOR Network), life science research (see the BioSharing Information Resource), or the ARRIVE guidelines for reporting work involving animal research. Where applicable, authors should refer to any relevant reporting standards documents in this form.

If you have any questions, please consult our Journal Policies and/or contact us: editorial@elifesciences.org.

Sample-size estimation

- You should state whether an appropriate sample size was computed when the study was being designed
- You should state the statistical method of sample size computation and any required assumptions
- If no explicit power analysis was used, you should describe how you decided what sample (replicate) size (number) to use

Please outline where this information can be found within the submission (e.g., sections or figure legends), or explain why this information doesn't apply to your submission:

No explicit power analysis was used. Figures report results from at least three independent replicates, as reported in the figure captions. Experiments using primary cells or mitochondria from mouse embryos were designed to minimize the number of animals used in accordance with local regulations and after approval by the Animal Care Committee at Sunnybrook Research Institute as reported in the methods section.

Replicates

- You should report how often each experiment was performed
- You should include a definition of biological versus technical replication
- The data obtained should be provided and sufficient information should be provided to indicate the number of independent biological and/or technical replicates
- If you encountered any outliers, you should describe how these were handled
- Criteria for exclusion/inclusion of data should be clearly stated
- High-throughput sequence data should be uploaded before submission, with a private link for reviewers provided (these are available from both GEO and ArrayExpress)

Please outline where this information can be found within the submission (e.g., sections or figure legends), or explain why this information doesn't apply to your submission:

Figures 1 and 9
(A) The cell based assays report mean values for 3 separate experiments with >400 cells analyzed for each condition. The means reported individually and as a means of means were assessed for significant differences using a one-way ANOVA within each group followed by a Tukey's multiple comparisons test. *p-values less than 0.05, **p-values less than 0.01, ns are non-significant p-values (>0.05) as reported in caption.
(B) Reports cell free assays using isolated mitochondria and purified proteins. Data from three independent experiments are shown as individual points, with lines representing the average. Some dots are not visible due to overlap as reported in the caption.
Figure 2
(B) Reports averages for three biological replicates each representing 90-1000 cells analyzed. The Bar height; mean. A one-way ANOVA was used followed by a Tukey's multiple comparisons test to compare the means of each group. * p< 0.05; ** p,0.01; ns p>0.05 as reported in the figure caption.
(C) Reports cell free assays using mitochondria isolated from embryonic day 15 (E15) mouse brains and purified proteins. Data from three independent experiments are shown as individual points, with lines representing the average. Some dots are not visible due to overlap as reported in the caption.
Figures 3,4,5,7

eLife Sciences Publications, Ltd is a limited liability non-profit non-stock corporation incorporated in the State of Delaware, USA, with company number 5030732, and is registered in the UK with company number FC030576 and branch number BR015634 at the address 1st Floor, 24 Hills Road, Cambridge CB2 1JP | August 2014

Statistical reporting

- Statistical analysis methods should be described and justified
- Raw data should be presented in figures whenever informative to do so (typically when N per group is less than 10)
- For each experiment, you should identify the statistical tests used, exact values of N, definitions of center, methods of multiple test correction, and dispersion and precision measures (e.g., mean, median, SD, SEM, confidence intervals; and, for the major substantive results, a measure of effect size (e.g., Pearson's r, Cohen's d)
- Report exact p-values wherever possible alongside the summary statistics and 95% confidence intervals. These should be reported for all key questions and not only when the p-value is less than 0.05.

Please outline where this information can be found within the submission (e.g., sections or figure legends), or explain why this information doesn't apply to your submission:

Where indicated in the figure captions the means reported individually and as a means of means were assessed for significant differences using a one-way ANOVA within each group followed by a Tukey's multiple comparisons test. *p-values less than 0.05, **p-values less than 0.01, ns are non-significant p-values (>0.05) as reported in caption. For other figures SEV was used to estimate error when the mean of means is reported and is indicated in the captions and methods.

(For large datasets, or papers with a very large number of statistical tests, you may upload a single table file with tests, Ns, etc., with reference to sections in the manuscript.)

Group allocation

- Indicate how samples were allocated into experimental groups (in the case of clinical studies, please specify allocation to treatment method); if randomization was used, please also state if restricted randomization was applied
- Indicate if masking was used during group allocation, data collection and/or data analysis

Please outline where this information can be found within the submission (e.g., sections or figure legends), or explain why this information doesn't apply to your submission:

Experimental groups were allocated on the basis of the proteins and/or cell line being analyzed as specified in the figure captions.

Additional data files ("source data")

- We encourage you to upload relevant additional data files, such as numerical data that are represented as a graph in a figure, or as a summary table
- Where provided, these should be in the most useful format, and they can be uploaded as "Source data" files linked to a main figure or table
- Include model definition files including the full list of parameters used
- Include code used for data analysis (e.g., R, MatLab)
- Avoid stating that data files are "available upon request"

Please indicate the figures or tables for which source data files have been provided:

I data is provided when the data was fit to calculate values reported. For example, apparent dissociation constants (Kd) in Figure 6 were measured from raw data in Figure 6 – figure supplement 1 for each binary interaction. Similarly the ANTS/DPX assay data used to measure activation of Bax (EC50s in Figure 6) is provided in Figure 6 – figure supplement 1.
